# From active duty to activism: how moral injury and combat trauma drive political activism and societal reintegration among Israeli veterans

**DOI:** 10.3389/fpubh.2024.1336406

**Published:** 2024-06-05

**Authors:** Adi Levy, Michael L. Gross

**Affiliations:** ^1^Department of Jewish Studies and Political Science, Florida Atlantic University, Boca Raton, FL, United States; ^2^Israel Institute and Florida Atlantic University, Boca Raton, FL, United States; ^3^School of Political Science, The University of Haifa, Haifa, Israel

**Keywords:** moral injury, morally injurious events, social activism, political participation, combat trauma, veteran integration

## Abstract

**Trigger warning:**

This article deals with combat experiences and their consequences and could be potentially disturbing.

**Introduction:**

Moral injury (MI) is a severe form of combat trauma that shatters soldiers’ moral bearings as the result of killing in war. Among the myriad ways that moral injury affects veterans’ reintegration into civilian life, its impact on political and societal reintegration remains largely unstudied but crucial for personal, community, and national health.

**Methods:**

13 in-depth interviews examine combat soldiers’ exposure to potentially morally injurious events (PMIEs) that include killing enemy combatants, harming civilians, and betrayal by commanders, the military system, and society. Interviewees also described their political activities (e.g., voting, fundraising, advocacy, protest) and social activism (e.g., volunteering, teaching, charitable work). Interviewees also completed the Moral Injury Symptom Scale.

**Results:**

Two distinct narratives process PMIEs. In a *humanitarian* narrative, soldiers hold themselves or their in-group morally responsible for perpetrating, witnessing, or failing to prevent a morally transgressive act such as killing or injuring civilians or placing others at unnecessary risk. In contrast, a *national security* perspective blames an out-group for leaving soldiers with no choice but to act in ways that trigger moral distress. Associated with shame and guilt, the humanitarian perspective triggered amends-making and *social* activism after discharge. In contrast, a national security perspective associated with anger and frustration fostered protest and intense *political* activism.

**Discussion:**

Despite its harmful health effects, moral trauma and injury can drive intense political and social activism, depending upon the narrative veterans adopt to interpret PMIEs. Aside from moral injury’s personal, familial, and social effects, moral injury drives veterans’ return to the political arena of civil society. As such, veterans play a central role in politics and dramatically affect post-war policy in democratic nations following conflict.

## Introduction

Moral injury is a potent and debilitating mental health crisis that combat soldiers face (1, 2). Moral injury, or more specifically, *military* moral injury, denotes the intense psychological distress following potentially morally injurious events (PMIEs) that comprise injuring or killing others in war. The distress is moral because killing, however convincingly sanctioned by the principle of self-defense, remains deeply disturbing. While moral injury may follow unlawful acts of rape and murder, it is far more pervasive in the wake of *permissible* killing in war, including killing enemy combatants or collaterally killing civilians. Shame, guilt, anger, and frustration accompany moral injury and distress, where the most severely morally injured may suffer debilitating anomie, self-condemnation, social withdrawal, and low self-esteem. Others, like the subjects of this study, undertake political activism and become essential participants in the public discourse about war.

Investigating social activism and political participation among Israeli combat veterans, this study describes how exposure to PMIEs affects subsequent reintegration into political life. Beginning with a literature review, we examine three distinct domains that rarely intersect: moral injury, political participation, and veteran activism. Turning to the results, the data demonstrate how reactions to PMIEs are far more variable than previously suggested. Some combatants adopt a humanitarian narrative and attribute their moral distress and subsequent activism to their shame and guilt from killing innocents in war, however legally defensible this may have been. But another group takes a different tack and utilizes a “national security” narrative to interpret morally injurious events. These combatants exhibit anger and frustration more than shame and guilt. Indignant at being forced to harm civilians by an enemy who uses human shields and fights without uniforms, these soldiers return to civilian politics to defend and justify their actions. Each group then, engages in a unique form of political activism, drawing from and relieving their moral distress. We take up the theoretical and practical implications of the data in our discussion.

## Literature review: moral injury, political participation and veteran activism

The literature review emphasizes the intersection of the three domains unique to this study: moral injury, political participation, and veteran activism.

### Moral injury and morally injurious events

Moral injury often occurs in extreme conditions of war where familiar moral norms collapse and cease to provide the necessary anchors of human behavior. These circumstances, usually conceptualized as potentially morally injurious events (PMIEs), may lead to moral injury (MI) and comprise incidents of “perpetrating, failing to prevent, bearing witness to, or learning about acts that transgress deeply held moral beliefs and expectations [(3): 695].” PMIEs embrace distinct incidents: perpetration and betrayal events. While perpetration events may include severe moral and legal transgressions (e.g., mass murder), most comprise ethically permissible acts of killing (e.g., self-defense). There are two categories of perpetration events. *Perpetration-by-self* (self-perpetration) denotes acts of commission (e.g., killing or injuring) by the morally distressed or injured combat soldier. *Perpetration-by-others* (other-perpetration) signifies acts of omission in the face of killing by members of one’s unit during an action where the morally injured soldier witnessed the act and/or was unable to prevent it (4).

In contrast, betrayal-based events occur when trusted authorities, such as military commanders or political leaders, pursue morally transgressive policies that betray or undermine the trust and faith soldiers have in these same authorities (5, 6). Betrayal may come when commanders or leaders issue orders that violate soldiers’ conceptions of ‘what’s right,’ abuse their authority, or take futile action that leads to needless death and injury (1). Circumstances may multiply and intensify PMIEs. These include leadership flaws, commanders’ failure to provide guidance in morally complex situations, indiscriminate or inhuman enemy tactics, and disruptive civilian-military relations. In the context of civil-military relations, soldiers may also construe the violation of their expectations from members of society as a form of betrayal (7). For example, soldiers could feel that despite their expectations, their compatriots do not appreciate the sacrifices they are making to protect them. In these circumstances, moral injury develops in those soldiers who feel profoundly betrayed by the same society that relies on them in times of war.

Shame, guilt, and anger drive moral injury (8). While many moral transgressions may precipitate guilt, the guilt related to moral injury arises when a person inflicts harm on another and then perceives themselves as a bad person (9, 10). In response, the guilty person often tries to remedy the wrongdoing by undertaking measures to minimize the damage caused (11). Related to guilt, shame arises following a moral transgression or demonstration of incompetence whereby one is shown to be inadequate (12), often resulting in feelings of worthlessness and inferiority (13–16) accompanied by attempts to hide or withdraw. Responses to guilt and shame may also lead individuals to acknowledge their or their in-group’s (compatriots’) responsibility, apologize or ask forgiveness, or offer reparation or compensation [e.g., (17, 18)]. Anger and frustration arise following experiences of unfairness, betrayal, or injustice. While frustration often generates anger, frustration is a distinct emotion when one cannot operate as he wants (19: 15–17). Both frustration and anger might trigger the motivation to attack, humiliate, and seek retribution against the agent of injustice. Frustration, anger, and retribution-seeking characterize the reactions of many soldiers when they face enemy threats or institutional betrayal [(1): 114, also (20)].

The potential of PMIEs to precipitate moral injury depends on their severity. A holistic view suggests that PMIEs cause moral distress because they compel agents to choose between two conflicting moral values when agents are forced into a choice incompatible with their moral inclinations (21, 22). The less compatible one’s actions, the more morally transgressive they become, and increasingly likely to cause moral injury (23).

Perceptions of a just world, personal beliefs about the goodness of self/others (3, 24, 25) , moral foundations, and other personality traits (26, 27), cause injurious events to develop into full-fledged MI. As a result, the moral distress resulting from PMIEs may range from moral injury accompanied by withdrawal, self-handicapping and depreciation, and self-harm to less severe but no less disturbing moral unease and anguish [(28, 29), for review].

Treatment for moral injury encourages self-forgiveness and compassion through cognitive-behavioral and other psychotherapies and proactive behavior characterized by making amends (3, 30, 31) and resilience training (32). In most cases, these treatments are directed by clinicians, but in the instances described here, they are self-directed and emerge as social and political activism to make amends.

### Morally injurious events, amends making, and social and political activism

In the wake of moral injury, “amends making” denotes personal behavioral changes that focus on social repair, “righting a wrong,” and allow veterans to “reconnect with their lives and feel like a contributing member of society” (3, 33). Within the constellation of emotions characterizing moral injury, guilt and, to a lesser extent, shame are the principal drivers of making amends (3). To expiate guilt and mitigate moral injury, affected veterans are often encouraged to formulate an “amends plan” or a work agenda that includes pro-social behavior such as devoting time to family, friends, and co-workers, visiting fallen soldiers’ graves, charitable work, community volunteering, or repartitions for those directly affected by one’s conduct in war (34, 35).

While guilt and shame encourage amends-making, anger and frustration lead one to seek justice and compensation (36). Frustration arises with feelings that external conditions have deprived persons of their basic entitlements. Frustration and anger motivate political participation and social activism to seek justice (37) or regain lost entitlements [(19): 15–18]. Participatory responses include protest activity and nonviolent civil resistance that, if unsuccessful, may lead to aggressive political participation, such as civil disobedience, violent demonstrations, and riots.

We suggest that organized social activism and political participation are an extension of amends-making, social repair, and other activities intended to change existing social structures at the domestic or international level. Social activism is distinctly pro-social and focuses on works designed to aid and improve affected populations, whether one’s own or another’s. Social activism embraces volunteerism, community engagement, education, legal aid, charitable work, and other service-oriented activities. In contrast, political participation addresses the locus of governmental or corporate power to affect change through institutional channels. Political participation includes voting, petition writing, mass protest, political advocacy, boycotts, and party organization (38).

While studies consistently show how military service strengthens political participation by conferring the necessary skills, networks, collective responsibility, and civic pride (39–41), the impact of combat-related moral distress and moral emotions such as anger, shame, and guilt is unclear. Commenting on the corrosive effects of betrayal-based moral injury, Shay (42) speculated that “unhealed combat trauma devastates the civic and political life of the returning veteran” for whom moral injury has “obliterated the capacity for trust,” rendering political struggle a “hollow charade.” But few, if any, studies have explored the relationship between combat-related MI, social activism, and political participation.

Nevertheless, the emotional components of combat-related moral distress overlap with social activism and political participation as individuals become socially and politically active to enhance personal growth and empowerment, increase self-worth (43), fulfill a sense of responsibility or mission, make a difference or leave a legacy (44). While guilt primarily drives amends-making, the extent to which guilt affects political behavior turns on the difference between individual and collective guilt and a careful distinction between political participation aimed at protest and social activism and actions geared toward melioration, restitution, and compensation.

Although individual guilt is not a common motivator of political action, protest, or online activism (45), collective guilt elicits feelings of blame and responsibility for injustices visited by one’s in-group on an out-group (through racial discrimination or military occupation, for example). Subsequent behavior is marked by restitution or compensation, civic engagement, volunteer work or collective action (46, 47). Each component of collective guilt-driven social activism is integral to amends making. Alongside collective guilt, however, are expressions of collective *pride* and *anger*. Taking pride in one’s in-group or nation’s achievements or confronting those threatening a group’s values, life, or property motivates political activism, ranging from protest, boycott, censure, and public advocacy to verbal abuse and physical attacks directed against opponents (48). The role of these emotions emerges in stark relief in the context of veteran’s social activism and political participation.

### Political participation among veterans

Except for Shay’s speculative remarks, no study investigates the relationship between moral injury and veterans’ subsequent political participation and social activism. Understanding that perpetration-based moral injury exacerbates feelings of shame and, particularly, guilt to encourage amends-making among affected veterans, we hypothesize a similar association between guilt, particularly collective guilt, and social activism characterized by charitable work, volunteering, community engagement, and the pursuit of social justice for the victims of soldiers’ actions in combat. In their study of Danish veterans returning from Afghanistan, Brænder and Andersen (49) explain how the dehumanization of Afghan civilians increases the motivation to serve society by entering public service. They do not describe moral injury but instead consider “the idea of making a difference for society as a whole (p. 474).” In this way, public service can be construed as a form of amends-making that aids compatriots at home instead of assisting those civilians harmed abroad during military operations.

At the same time, however, betrayal-based moral injury arouses anger and frustration that may be directed toward two sources: compatriot military organizations or domestic or foreign institutions perceived as threats. In the first case, soldiers may feel betrayed by their military superiors or political leaders for poor decision-making, moral fecklessness, abuse, unnecessary death and injury to compatriots and enemies, and negligence toward veterans. Among 1960s Black veteran activists, Parker (50) highlights deep-seated grievances stemming from racial discrimination during military service and an unfulfilled sense of entitlement to civil rights and equality upon returning home. Intense feelings of betrayal and anger directed against the military and government drove conventional but dangerous forms of political participation, such as voting and attending political meetings. Schrader’s (51) account describes how veterans demilitarize and “reinscribe” their patriotism to protect their nation by pursuing a social justice agenda by engaging in antiwar and anti-corporate protests (e.g., Occupy Wall Street). There is no reason, however, that conservative-leaning veterans might similarly redefine their nationalism to defend entrenched military and political agendas. Here, betrayal comes from without, from domestic organizations or foreign governments who disparage a nation’s armed forces or military campaigns. In both instances, anger rather than guilt or shame is the dominant response to betrayal MI and a motivator of political action aimed at refuting prevailing criticisms of wartime killing. Building on these arguments, the data offered here suggest that distinct experiences (perpetration or betrayal events), culpable agents (self, other, military, foreign or domestic), and emotions (shame, guilt, anger, or frustration) affect how soldiers interpret morally injurious events and take up subsequent social and political activism as they reintegrate into civilian life.

## Methodology

This study investigates the relationship between PMIEs, the intense emotions they elicit, and political participation among 13 politically active veterans. Our sample, *n* = 13, corresponds with studies that suggest that 6–12 interviews suffice to identify categories (52, 53).

### Participants

Twelve of the 13 participants were male. Our participants were social and political activists who served in an infantry or combat unit interacting with the Palestinian civilian population. Participants were at least one year after their discharge. Participants’ ages ranged from 25 to 49 at the time of the interview, with a mean of 33.5. All interviewees served as reservists in the Israel Defense Forces (IDF) and were politically or socially active at the time of the interview. Interviewees were engaged in advocacy, raising awareness campaigns, protests, educational projects, and online activity. Interviews were conducted between 2020 and 2021. As the research began during the COVID-19 pandemic, interviews were conducted via Zoom using a snowball sample. Snowball sampling identifies index individuals, collects information from each, and asks them to refer others suitable for the study. Snowball sampling is best suited when subjects are difficult to reach, or a sufficient sample is unlikely to be obtained from random sampling (54). This method enabled us to confirm respondents’ social and political activity. Before the interview, respondents completed the Moral Injury Symptom Scale (55). They were then invited to an open conversation where they were asked to share their military experiences and describe how these experiences influenced their choice of becoming politically and socially active. They were not introduced to the term “moral injury” or PMIEs and were only asked to share, “What do you take from your military service? Is there something you would have done differently? Are there any particular incidents that you remember affected you?” In their responses, they brought up incidents that left their mark. The University of Haifa ethics committee approved this research.

### Analysis

To unpack moral injury constructs, quantitative and qualitative studies often build on semi-structured interviews (24, Williamson et al., 2020) (1). As a qualitative study, we opted to use in-depth interviews and grant complete freedom to participants. This enabled us to learn about their motives and behaviors and let interviewees feel comfortable sharing their experiences (56, 58). Open conversations further allowed us to identify each respondent’s perspective to interpret wartime events (59, 60). Interviews were collected and transcribed, and interviewees’ names were anonymized. The names that appear below are random names assigned to respondents to ensure their privacy. We translated the interviews from Hebrew to English with minimal editing for clarity.

The analysis was conducted by the authors, we divided the data into two categories. (1) A humanitarian perspective in which respondents placed blame on themselves or their group for the morally transgressive events we identified. (2) A national security perspective in which respondents placed blame on the enemy for the transgressive event. Then, we coded data for PMIE types, including self-perpetration, other-perpetrated events, and betrayal events.

PMIEs give rise to various affective and cognitive states among soldiers experiencing these events. Affectively, soldiers may exhibit intense guilt, shame, anger, or empathy, as they interpret events in ways ranging from military necessity and defense to domination, exploitation, ingratitude, and futility. Disparate PMIEs coupled with differing emotions and event interpretations precipitate motivations for political and social activism, including making amends, exercising social responsibility, or demonstrating national commitment (protest, volunteering, aiding victims of war) and social responsibility (lobbying, education). These factors—PMIE type, affective and cognitive states, affect the motivation for participation and social activism, including public protest, legal advocacy and lobbying, education, and volunteer work.

## Results

The results emphasize two complementary sets of findings. The first finding broadens the spectrum of morally injurious events to embrace two narratives of morally injurious events drawing from humanitarian and national security concerns, respectively. Each reflects disparate emotional attributes ranging from guilt and shame to anger, frustration, and pride, the latter hitherto unnoticed in accounts of moral injury. Six interviewees adopted a humanitarian perspective. Four interviewees adopted a national security interpretation of events. Three more expressed ambiguous feelings and swayed between a humanitarian and a national security perspective. The second discussion illuminates how moral injury and distress coupled with each narrative drive political participation and social activism among veterans.

### Two narratives of morally injurious events

During their military service, the interviewees engaged enemy combatants, conducted house searches, guarded checkpoints, and patrolled contested territory. Despite the similarity of events, interview data reveal two prevailing narratives—“humanitarian” and “national security”—veterans adopted to interpret their experiences. Table 1 shows the division of interviewees’ perspectives.

**Table 1:** Division of interviewees’ PMIE and perspectives.

	Event(s) described	Interpretation
Dan	Other-perpetrated	National security
Alon	Other-perpetrated	National security
Tal	Other-perpetrated	National security
Ronny	Other-perpetrated	Humanitarian
Moshe	Other-perpetrated/betrayal	Ambiguous
Oren	Other-Perpetrated/betrayal	National security
Gal	Self-perpetrated/other-perpetrated/betrayal	Humanitarian
Yossi	Self-perpetrated/other-perpetrated/betrayal	Humanitarian
Guy	Self-perpetrated	Ambiguous
Nir	Self-perpetrated	Ambiguous
Eyal	Self-perpetrated	Humanitarian
Aviv	Self-perpetrated	Humanitarian
Eran	Self-perpetrated	Humanitarian

The humanitarian narrative draws from the intense moral and psychological difficulty of killing in war. In this narrative, veterans hold themselves or their in-group (e.g., nation or society) morally responsible for perpetrating, witnessing, or failing to prevent a morally transgressive action such as killing or injuring civilians, violating human rights, or placing others at unnecessary risk. Feelings of disproportionate and indiscriminate killing as part of an oppressive enterprise of military occupation over civilian society intensify moral distress and apprehension. In contrast, the national security narrative draws on self-defense and blames an out-group (e.g., their adversary or a third party) for leaving combat soldiers with no choice but to act in ways that trigger moral anguish. In this view, Israeli military control over the West Bank is an essential component of Israel’s struggle for existence. National security-oriented veterans expressed a keen sense of mission. Their moral agony was the result of being forced to act in a morally contentious way as a reaction to the enemy’s desire to destroy the Jewish state through unrelenting terrorism and unlawful warfare (e.g., human shields). Thus, when describing their experiences, these veterans focused on the harm they strive to prevent despite the complex circumstances on the ground.

#### The humanitarian narrative of PMIEs

Confronting self-perpetrated, potentially morally injurious events, other-perpetrated events, and acts of betrayal, interviewees adopting a humanitarian narrative to interpret their experience immediately reveal intense conflict. On one hand, they describe their motivation to join the army to defend their country. On the other, their motivation to fight was tempered by extreme moral disquiet because circumstances demanded that they operate in ways that violated their inner moral compass. Each action or event reveals a distinct phenomenology.

##### Self-perpetrated actions

Self-perpetrated actions comprise shooting at enemy combatants or other military targets, utilizing violence, conducting house searches, and searching civilians unnecessarily. Here, the most severe acts required lethal force and included the killing of others. Feeling reluctantly placed in these situations, some interviewees took full responsibility for the harm they inflicted on the “enemy.” These circumstances triggered moral turmoil as some interviewees felt like “thugs,” believing their actions “corrupted their moral consciousness,” and realized their actions were unjustified. Eran, a sniper participating in an ambush to capture a suspected terrorist, describes his apprehension:

“I told myself, this is crazy. This man is smoking a cigarette and does not realize he has a red dot on his head. In another second, he’s going to die. You know, you are looking at a man’s last moments, not in a movie. It is reality, and you are doing it. Immediately after we killed him, there is a second of joy. We did what we came to do, but it’s also very, very frightening. […] and all the while, there’s the thought that we killed a human being. That’s a disturbing thought.”

This description focuses on the moral transgression of violating the humanitarian, moral code prohibiting killing. Typical of the humanitarian narrative, it pays little attention to the circumstances that led to killing a person, any appeal to self-defense, or a cost–benefit analysis that might justify or defend the outcome.

Alongside killing, more moderate experiences such as chaotic and harshly conducted house searches also triggered moral distress. House searches require soldiers to forcibly enter a home, often very late at night, to search for terrorists or interrogate the house members to identify potential collaborators. Confrontations between soldiers and civilians quickly turn nasty. Moral anguish grows as soldiers exercise authority over an enemy viewed as weak and vulnerable and, as a result, perceive the searches as increasingly unjustifiable. Ronny, who often joined military operations, described such a case:

“In our case, we did not enter the house. We just told them to come out to the entrance. And then, I saw the children. I could see that they hated us, and rightly so. I mean, we are earning that hatred. The house I remember most was one with three children aged 3–10. I told myself that I am raising the next generation of terrorists here because I am teaching them that Israelis are people with rifles and helmets and loaded with military equipment, while they [the children], by definition, were innocent. I think that I felt guilty and most of all, I just did not want to be there. I did not want to be part of this thing.”

As in the previous depiction, the interviewee overlooks the mission’s necessity (incapacitating the enemy) to focus on the moral burden of frightening children and humiliating their father in the middle of the night.

##### Other-perpetrated actions

In other-perpetrated actions, soldiers witnessed their unit’s members performing morally questionable acts they could not prevent. Gal, witnessed military operations firsthand and describes such an event:

“There is an element of “we will educate them; we will punish them. They will learn a lesson.” There was a case where a Molotov cocktail was thrown on the road. And someone gave instructions to close all the shops, which is like shutting down Allenby Street [a main thoroughfare] in Tel Aviv. They [Israeli soldiers] shout on the loudspeaker and throw stun or tear gas grenades. In one case, our officer sounded amused. He told everyone that his soldiers threw a stun grenade, and everyone ran out like mice. I felt a lot of anger. I thought I was the only one feeling that way, so I did not speak up. I did not share because it is clear what the social codes are.”

Responding to the question, “How did that make you feel,” Gal said, “I was ashamed for being there; I could not do anything. Gal’s description contains two essential elements of soldiers’ experiences. First, it focuses on the disproportionate and indiscriminate use of power. Second, the soldier emphasized the central role of internal social codes regulating soldiers’ relations with one another. Soldiers often avoid sharing their emotional distress because they fear a social backlash. Harboring these feelings amplifies their grievances. Apart from dealing with the moral discomfort of the situation, they feel misunderstood, out of sync with others, alienated and excluded, and left with no choice but to remain silent. This case also demonstrates how perpetration-by-others intertwines with betrayal. Gal’s moral distress stems from not speaking up in the face of moral transgressions by others. But Gal also hints at betrayal by comrades, trusted commanders, the army, and society.

#### Betrayal

Betrayal describes broken trust in close relationships. Combat soldiers place significant trust in their commanders or comrades-in-arms. Therefore, betrayal from trusted commanders may bring profound and debilitating harm to the person betrayed. Moshe, described this broken trust in one operation during which his commander jeopardized the entire team.

“In one operation, I was astonished by the lack of ethics in the unit I was assigned. And I warned about it at every possible opportunity. From the beginning, I noticed the unit commander’s lack of transparency; he did not tell his superiors the actual state of readiness of the force. After preparing for one operation for ten months, I knew that there was no chance of carrying out the mission successfully. I saw how the commander lied to his superiors, falsely reporting that the force was prepared. I saw how soldiers around me remained silent in the face of these lies, and I had a very, very, very hard time with it. We had a personal conversation with the head of the intelligence unit. He knew there were problems, and I told him I had no idea why we were going. The commander is simply lying to his superiors. In retrospect, I realized the head of Intelligence asked the unit commander to stop the operation. He did not stop it. And our force was exposed, and we had a very difficult encounter with enemy combatants.”

Despite knowing this, neither Moshe nor other unit members confronted their commander. Interestingly, while this description can easily be viewed as a tactical mistake by the unit leadership, Moshe emphasized the lack of ethics, trust, and transparency and blamed his commanders for irresponsibility, incompetence, and moral dishonesty, which triggered acute moral distress.

Yossi, described the loss of trust in the system:

“My commander said, let us use the opportunity. We have extra MAGAV (Border Police) teams that entered the village with us. We can join forces and open a checkpoint between two Palestinian villages. I said OK, but why? The commander replied that Palestinian drug dealers are using this route, and we want to show them that we are here. He instructed us: “Stay there for two hours and leave.” It was the middle of the night, it was dark, we could not see anything, and were opening a checkpoint and waiting. A truck passed, and we stopped it for inspection. No one told us what we were looking for. And then, there was another car with a family, and it suddenly hit me. I remember thinking, I am risking my life, but I am not defending Israel. I tried to remember why I got drafted. To defend Israel and defend my family and friends, and I just cannot see the connection—I know for a fact there’s no connection.”

While describing this incident, Yossi’s anger was glaring. He blamed his commander for risking his life unnecessarily. His thoughts focused on how the actions he was required to perform conflicted with his motivation to join the army, a contradiction that triggered feelings of broken trust in the system and betrayal. He felt he was lied to and forced to perform a mission unrelated to the country’s security.

This section shows how a humanitarian interpretation of events triggered intense moral discomfort characterized by shame, guilt, embarrassment, and remorse. In contrast, soldiers adopting a national security narrative acknowledge the significance of their duties and the necessity of their actions. However, they suffer from anger and frustration with the situation they are forced to confront.

#### The national security narrative of PMIEs

In the national security interpretation group, the national security narrative focused on successfully prosecuting a complex war on terror. Here, soldiers appreciated their mission’s complexity but blamed the enemy (the out-group) for the unlawful use of human shields, attacks on civilians, failure to wear uniforms, and abuse of medical vehicles that compelled the Israeli soldiers to use deadly force and endanger civilian lives. Frustration with the enemy’s unethical and unlawful tactics that endangered soldier’s lives, and anger from criticism and charges of excessive force were the dominant emotions interviewees displayed. Frustration and anger played out in self and other-perpetrated actions and betrayal.

##### Self-perpetrated actions

While using force remained morally troubling, participants who adopted the national security narrative were primarily concerned with complying with their professional duties, completing their mission, protecting their fellow combatants, and adhering to the military code of conduct. In the following case, Dan describes how NOT opening fire triggered moral distress because it meant risking his team members.

“I had a situation where I had a target (a terrorist) in my sight. I knew how to hit a “target” from 100 meters, but there was the risk of people being hit nearby. I chose not to shoot but to ask for permission, and I did not get permission. The Major General was on the other end of the line. He asked me, ‘Are you sure you can hit from 100 meters without hitting civilians?’ I can never be 100% sure. He told me, ‘So I do not approve.’ It was not an easy decision for me because, in real-time, you never know if your friend will get shot and killed by the enemy. You’re supposed to respond to fire. It is the IDF’s leading value, *perseverance in the mission, and dedication to the pursuit of victory*. That is a core value!”

This description offers an extraordinary example of broken values rarely addressed in MI literature that usually focus on killing as a morally transgressive action. Here, the potential implications of not shooting became a source of moral distress for Dan because it meant that he was not doing his job, violating the values he adopted as a soldier, and risking the lives of his friends.

Incidents like house searches described earlier were also mentioned by soldiers who adopted a national security interpretation. In these cases, soldiers acknowledged the difficulty of entering a house in the middle of the night, but they viewed their actions as necessary:

“You gather all the members of the house after scanning the house itself. The house is not chosen because its occupants were incriminated but because of its location with a view over vital territory. For example, you can go into a house because you have information about a smuggling tunnel that you can watch over from that house. So, you occupy the house to protect your comrades working in the tunnel. But you terrify a family that had done nothing wrong. They are innocent, as far as you know. There is no reason for them to suffer except that they live in a house that is essential to completing the mission.”

Dan commented on this experience saying that while it was an unpleasant situation, he did not suffer any guilt—“I know why we were there, and I knew we did the best we could.” Placing the mission at the forefront diverted attention to the enemy’s actions, thereby mitigating feelings of guilt. Nevertheless, focusing on the enemy’s behavior to interpret events triggered deep frustration for being entangled in situations where soldiers were unable to “win.” The following description by Nir, illustrates the moral discomfort soldiers might experience when exercising authority.

“One rainy night, I caught a young boy who was trying to smuggle eggs. He had a cart full of eggs. I turned to the agricultural unit responsible for coming to pick up such smuggled merchandise, but they did not want to come because it was the middle of the night. They told me that the eggs must be destroyed. They told me that I was not allowed to destroy the eggs; the kid had to destroy them himself. I explained to him assertively that he must destroy the eggs. He started crying and breaking all the eggs on the road. I remember it vividly. I stood there with my rifle pointed toward that poor kid until he destroyed all his eggs. I think he had thousands of eggs there. I hated it. It was awful and so frustrating. Specifically, I hated my subordinate soldiers, who were so insensitive.”

Nir felt it necessary to explain that he had no other choice and knew his actions were necessary. He explained that if he let the kid go on with the merchandise, it would have gotten to Israel without proper inspection and could potentially become a health risk. At the same time, he thought that the kid would probably be beaten when he got home for losing all the merchandise. These incidents match the PMIE in which one is forced to perform a morally disturbing act. Nir’s story could easily fit the humanitarian narrative, but he justified his actions with national security obligations. His testimony illustrates the moral struggle soldiers face when their commitment to national security conflicts with their humanitarian principles. In this case, Nir’s commitment to national security won over his humanitarian inclinations as he forced a child to destroy all the merchandise he was carrying.

##### Other-perpetrated actions

From the national defense narrative, the concepts of perpetration-by-others and betrayal differ markedly from the humanitarian narrative. Among those viewing events through the prism of national defense, the ‘others’ in other-perpetrated actions are the enemy. Dan, for example, describes his pride at accomplishing a difficult mission further imperiled by the enemy’s “dirty” tactics:

“Generally speaking and considering the highly complicated challenges facing the IDF how the enemy used civilians as human shields and exploited UN facilities, ambulances and the Red Crescent symbol for terrorism, I think we can be *very proud* of ourselves and very proud of the way we handled the situation.”

For participants who mentioned these actions, the enemy’s odious conduct put their unit members at risk and left little room for remorse or regret for their behavior during combat. However, these participants felt that the military code of ethics sometimes tied their hands as they fought an enemy trying to kill them and their friends. Alon, described:

“We were constantly engaged in arresting terrorists, you know, terrorists with blood on their hands. We arrested the head of Hamas in Qalqilya and Tukaram in complex operations. Many stones and rocks were thrown at us. We often endangered ourselves. Often, the team risked going into alleys. Even though we had a clear indication (of where the suspect was) and it was possible to shoot him from the air, we did not because it was a dense urban neighborhood. I’m very proud of how we handled these situations, which, to begin with, is a shitty situation.”

In these cases, the enemy commits acts that the Israeli soldier defines as morally transgressive. Structurally, these acts are no different than those described by the humanitarian narrative. In each instance, an enemy or commander violates the soldier’s deeply held moral convictions. However, the soldier’s ability to maneuver or respond in each case vastly differs. When confronted with a commander who lied and risked the lives of his troops, Moshe castigated himself for remaining silent and suffered as a result. Alon and Dan, however, cannot speak out and rebuke their enemy. All they can do is conduct themselves in ways that create moral distress by needlessly endangering their comrades. While expressing a solid commitment to the army and the country, soldiers who interpreted events through national-security narratives were, therefore, not immunized against moral unease, agony, and distress.

##### Betrayal

Viewing events through the prism of national defense, interviewees also displayed a unique sense of betrayal. Betrayal is commonly the result of fractured trust in commanders or political leaders. Among many national security respondents, however, betrayal reflected criticism by local civilians or the international community. Public reactions may trigger feelings of betrayal when soldiers feel their sacrifice is unappreciated and their actions harshly criticized. While the IDF enjoys widespread legitimacy in Israel, soldiers sometimes face criticism from citizens from different social sectors. As Oren, recalled:

“One time, I returned home after being away for two weeks. I lived then in an ultra-Orthodox neighborhood in Haifa. Some people started throwing stones at me. I ran after them to the synagogue, went to the rabbi and told him, “Have you no shame!?” I get back home after two weeks defending this country and getting stoned [by the enemy] every day and come back here to my house to be stoned by my own people. YOU are fighting ME? I am here for you. It is very, very, very difficult. It’s terrible. It was one of the hardest experiences of my life. The least you expect is that when you return home, people will smile at you and that you will feel loved.”

There is a subtextual description of social betrayal here, mainly due to Oren’s deep commitment to his country. This commitment created an unspoken expectation not fulfilled by one segment of society and intensified his emotional backlash. As he acknowledges, “It’s one of the hardest experiences of my life.” In 2017, the case of Eleor Azaria, a young combat medic who violated the rules of engagement and humanitarian law when he shot and killed a wounded and unarmed terrorist suspect generated a similar sense of betrayal. Although Azaria was tried and punished, there was sharp criticism from some segments of the public who berated the military, the press, and international rights organizations for their lack of support for soldiers sent to risk their lives for their country (61).

It is important to note that the neat division offered here is not dichotomous. Interpretations of morally injurious events were not entirely consistent. Some interviewees blamed their commanders for betrayal but acknowledged the value of their military service. Others saw their actions as necessary but still felt disquieted about the circumstances and reality imposed upon them. While the humanitarian and national security narratives described here might easily fit left and right political narratives, data from interviewees suggest that left and right narratives are an over-simplistic pattern to characterize the moral conflict soldiers might experience. Sometimes, left-wing veterans voiced their patriotism and concern for their country’s national security, while right-wing veterans expressed humanitarian concerns.

To investigate how interviewees’ expressions of moral distress mapped onto clinical measures of moral injury, 11 of 13 interviewees completed the Moral Injury Symptom Scale, Military Short Form (55), a 10-item scale to symptoms related to moral injury. The MISS registers moral injury on a scale of 10–100 (each question 1–10); The higher the score, the greater the degree of moral injury. Average scores were lower for national security, higher for humanitarian, and mid-range for the mixed narrative (27 [*n* = 3]; 43 [*n* = 5]; 31 [*n* = 3]).

While the humanitarian narrative scores approach the US average,^1^ the national security respondent scores reflect lower levels of scale-measured injury. Drawing on the interview data, we suggest that those espousing a national security orientation toward PMIEs suffer a different kind of moral trauma that existing moral injury scales do not measure entirely.

Despite disparate narratives and dominant emotions, the interviews highlight a clear direction of interpretation. Interviewees either blamed themselves or others for their morally contentious actions during combat. Self-blame triggered shame and guilt for committing or failing to prevent moral transgression. Other-oriented blame triggered anger and frustration. Anger arose when unlawful and unethical enemy tactics compelled soldiers to act in morally controversial ways. Frustration arose with being misunderstood by compatriots and delegitimized for their actions. These interpretations and the subsequent emotions shaped these veterans’ social activism and political participation as they return to civilian life.

### From morally injurious events to political and social participation

Humanitarian and national security narratives shape Israeli political discourse and affect soldiers’ perceptions and, indeed, their entire military service when they confront potentially morally injurious events on the battlefield. Following discharge, interviewees reveal how their interpretations of morally injurious events encourage political participation and social activism. The following section is divided into two parts that explain how humanitarian and national security interpretations of morally injurious events motivate social activism and political participation.

#### Humanitarian narrative activism (1): making personal amends

Social activism brings a sense of self-esteem, empowerment, and well-being that activists derive from assisting others and contributing to society (56). We address these personal benefits as mechanisms of self-compensation because they elevate social activists’ moral standing, allow soldiers to rebuild what their military service had broken, or help them justify their actions to themselves or others.

Following his discharge, Ronny—who participated in house searches and had many interactions with the Palestinian civilian population—currently engages in dialogue initiatives between Israelis and Palestinians, operates educational activities, collects testimonials from combat soldiers, and conducts online activity against Israel’s presence in the West Bank—illustrates this point.

“I felt that I had broken something, and now I needed to fix it. I was part of this system of the army whose idea was to oppress people, and I wanted to be part of the effort to fix and end the occupation… (Through my activity) I feel that I have returned to the experience of the army and rectify it, and this motivates me the most.”

In this case, the “broken something” recalls morally injurious events. In another instance, the triggering event was betrayal. Moshe, who earlier described his commanders’ betrayal, explained how feelings of social responsibility motivated him to demonstrate leadership to compensate for his inability to protest his commanding officers’ morally contentious orders.

“I believe that a person’s happiness is measured by his loyalty to his values. I measure myself and the people around me by their courage. For me, courage is how much a person adheres to his inner truth in a chaotic environment, and I think that was what motivated me. I felt that I had to stick to my truth. After I saw the dishonesty in my unit, I struggled to uncover it (after following orders). I stood up to the immense pressure from my peers and my superiors in the military to reveal the truth, and that made me feel brave. Similarly, when I stand up to all the social pressure in civil activism, it makes me feel brave.”

In this case, Moshe described how betrayal by his superior commander motivated political activism by allowing him to demonstrate his courage to stand up and struggle for his values.

#### Humanitarian narrative activism (2): consciousness raising

A sense of personal responsibility was another prominent theme. Taking responsibility for military actions that contributed to Israel’s ongoing occupation of the West Bank, some veterans turned to anti-occupation activism to alleviate the moral burden they carried from the disturbing events encountered during their military service. Asked if he felt responsible for the condition of the Palestinian population, Yossi responded:

“[I have] direct responsibility – that’s how I feel. Not only because I am a citizen of the country responsible for these violations, but today, I look at it mainly from the perspective of the Palestinians. For me, the most serious issue here is Palestinians’ human rights. I think this is my direct responsibility, both because I am a tax-paying citizen and because I know [the Palestinian living conditions]. Whoever knows this bears responsibility, and I was also a part of it. The combination of these makes it something I just cannot help but act against. In my view, I have taken part in something that I do not agree with in any way, and the least I can do is fight against it and use my advantage to try to turn it around.”

Those such as Ronny, and Yossi, who interpreted their experience as perpetrated by their in-group and shared their shame and guilt for being part of their occupying force, were motivated by a desire to change Israeli society from the inside and end the occupation. Respondents emphasized their need “to do something for society,” rectify the harms they witnessed or inflicted, and take responsibility for the ongoing occupation to which they contributed. Their activism comprised publicly sharing their combat experiences, conducting frank dialogue with future draftees, and sharing their military experiences online. While these activities might express fierce social criticism and anti-government activity, activists explain how they perform these activities on behalf of Israeli society. For example, Eran, who shared his experiences as a sniper and held a very critical stance on Israel and IDF, said:

“I do it (activism) to repair the place where I live. To some extent, I am an Israeli patriot. I have a very hard time with Israel. I do not sing the national anthem. The flag makes me sick, but I want to fight for this place because this is my place, this is the one piece of land in the world I am part of and a society that I am part of. Although it is a sick society, I need to fix it. The Israeli Zionist society is founded on a racist concept. At its core, Zionism has amazing precepts, but it is a romantic and social movement that attained this piece of land through colonialism. And basically, we are sick because we think we deserve more… I feel that I have a responsibility because of what I did in the service but also because of how I grew up. I had a wonderful family and an incredible childhood. Today, I say that since I have so much, I must give something back.”

This description depicts a stark paradox highlighting the wide variation of emotions that motivate veteran activism in Israel. Very often, anti-occupation activism elicits public backlash, particularly in Israel, because many Israelis object to anyone interpreting IDF actions as anything less than militarily necessary for Israel’s survival. Any other perspective is to distort reality. However, this interviewee explained how his activity was motivated by a social commitment *to* Israeli society, the same society that accuses many activists of undermining its social fabric. This paradox appeared in many interviews with activists who blamed their in-group for the moral transgressions they experienced and now engage in social and political activism critical of Israel.

In his interview, Aviv shared how he used excessive force against the civilian population during his service just because he commanded more power than the local inhabitants. He emphasized how he grew up in a violent society that influenced his behavior during the service. When asked about the motives for his online social activism against the occupation and his participation in high school educational initiatives, he answered:

“I want to change something in Israeli society and know what I want to change. Because I grew up in a very violent environment. I know what I want. It is not about a Zionist leftist ethos—The people I grew up with in my neighborhood were people who went to beat Arabs on Holocaust Memorial Day. I know that these people were not bad people. It’s simply the Israeli climate. If I want to change something, it’s that people do not grow up into something like that.”

Aviv expressed worries about his own society and adopted a critical approach that he hopes will awaken his society’s consciousness about the government’s policies towards Palestinians. Feelings of social responsibility also urged activists to engage in domestic protests against the government’s policies as part of one’s civic duty.

#### Humanitarian narrative activism (3): public protest

Veterans perceived social activism as part of their civic duty to maintain Israel as a democratic state. Emphasizing this sense of civic responsibility, some interviewees believed their activism was a social expectation of military service. Complying with this expectation was rewarding for these activists. Moshe, who served in an elite unit and previously described his feelings of betrayal, said, “Because of my role in the military, I feel that it is expected of me to continue this role through social activism. In being active and fighting and dealing with a chaotic social reality, I demonstrate courage. It makes me feel good. It feels fulfilling.”

Each form of activism is a reaction to combat-related morally injurious events and their subsequent interpretation via a humanitarian or national security narrative prism. Viewing their combat experiences through a humanitarian lens, activists laid blame upon themselves and their in-group. To expiate the resulting shame and guilt, veterans undertook social and political activism to make personal amends and raise awareness about the injustices perpetrated by the army through educational activities, online discourse, and public protest. In contrast, veterans adopting a national security narrative blamed the out-group, that is, the enemy, for the immorality of their wartime behavior. Combining social responsibility with a commitment to justify rather than atone for the morally transgressive acts they, their colleagues, or their government perpetrated, these veterans engaged in public advocacy at home and abroad. Their activism brought them to refute attempts to delegitimize Israel, expose the enemy’s unlawful and unethical military tactics, justify Israel’s military strategy, foster patriotism through education, and boost the country’s good name. In each case, veterans hope to set the record straight in the face of defamatory accusations.

#### National security activism (1): neutral information advocacy

Hoping to present a more balanced view of Israel’s military policy, some veterans adopt a neutral stance. Rather than vigorously advocating for Israeli policies directly, they chose to share their unvarnished combat experience. When asked about their decision to forego forceful advocacy in favor of publicizing his personal impressions, Tal described:

“There are two main reasons [for this decision]: first, I am not a psychologist, but I think it’s human nature: you want the person in front of you to understand your experience so they can understand you. Second, it seems to me that many times people share their experiences because they believe that talking about them will bring about a change in other people or political change. I share an experience from my military service with the expectation that someone will behave differently or that he will change his opinions. On the other hand, we also want to encourage people to be politically active. At the political level, it becomes much more complicated.”

This testimony illustrates how activists seek to explain the difficulty and ambiguity of their combat experiences. Tal shared these by referencing an incident he had at a checkpoint at the height of the Second Palestinian uprising (2000–2005). He described how he saw a child carrying a bag. His partner stopped the child for inspection and saw a ticking bomb inside the bag. Tal said that instinctively, he felt an urge to protect the child he thought was no more than 7 years old. He shared the conflict between the urge to protect the child and the anger of being placed in this situation. He emphasized how speaking about his military service enabled him to convey the complexity of the situation, which, he claimed, is often oversimplified and erroneously judged by others:

“I share because I want people to see the moral dilemma. I do not share because I’m looking for approval because I am very comfortable with what I did. And if I had made the wrong decision, the results would probably have been different. I want people to understand how complex it is. It’s not black and white.”

#### National security activism (2): pro-active information advocacy

In contrast to neutral advocacy, pro-active advocacy reinforces a national security agenda to unmask Israel’s enemy’s “real face” and to enlist international support. Rather than straightforwardly making amends, activists advocate on behalf of Israel. Anger and frustration spur political activism to contest unfair judgments, enhance understanding of Israeli policy, or confront the hypocrisy veterans see in some progressive social circles. Veteran activists undertook proactive information advocacy as part of the same feeling of civic responsibility that sustained their military service. Dan described how he risked the lives of his friends to spare the lives of innocent civilians by avoiding collateral harm during combat. In civilian life, Dan engaged in pro-Israel advocacy in Europe and the US. He describes his motives as follows:

“There’s this anger that motivates you. You know how things work and see how you are depicted by external spectators (e.g., foreign journalists, diplomats, and politicians). So, my anger was against these people who distort reality without understanding the real situation we faced as soldiers.”

External spectators’ moral judgment infuriated some respondents who interpreted morally injurious events as the enemy’s fault. These activists often engaged in political advocacy to defend the Army’s actions and express national pride. One interviewee, for example, said:

“I have no problem with what I did, but it angered me when people from the outside criticized me as an aggressor while overlooking the aggressive and manipulative actions of the other side. For this reason, I felt it necessary to expose their true face and advocate for the justice of our cause.”

Explanations of Israel’s actions range from clarifying the terror threats Israel faces to revealing the enemy’s combat tactics of using children as human shields, taking cover in civilian houses, and hiding ammunition in medical vehicles and UN facilities. As a combat soldier, Alon described a military operation to capture a suspected terrorist who was hiding with his wife and children. Responding to how the situation made him feel, he said: “You realize that you are at an inherent disadvantage despite your weapons because you are bound by specific moral codes, and your enemy takes advantage of this. In his civilian life, Alon conducted pro-Israel advocacy and volunteered to aid Holocaust survivors. He describes his activism as a civic duty:

“It’s simply the dissonance between how we are perceived compared to what I and my unit did. So, I think advocacy is a civic duty because this activity (advocacy) benefits the State of Israel. For example, if I come to a university campus to prevent students from passing anti-Israel regulations or participate in a debate, and the audience gets to hear from a former combat soldier and a commander, they might change their opinion. I have a chance to influence the future generation here.”

#### National security activism (3): patriotic education

Political advocacy is also conveyed as an educational endeavor. While advocacy primarily targeted an international audience, patriotic activists also sought to raise awareness within Israeli society. Activists initiated lectures, appeared in high school classes to encourage students to serve in combat units, provided data about the origins of the conflict, and offered accounts of their military experiences.

“I initiated a project in my high school. I got into every classroom and talked to students. I explained to them how to get to every unit in the Army and why it is worth going there. I described the best units and the experiences they will have wherever they serve. Two years later, I learned that the school had the highest enlistment rate in combat units in the school’s history.”

While it is difficult to determine whether the activists successfully encouraged students to serve in combat units, the description of his advocacy demonstrates the close connection between serving in the army and persuading others to serve in the Army.

## Discussion

As they serve in combat, soldiers confront potentially morally injurious events (PMIEs). The emotions these events evoke during and after military service and the subsequent kind of activism they undertake are mediated by the narratives they utilize to interpret combat trauma. The discussion engages four topics: (1) how morally injurious combat events, multiple narratives, and their attending emotions spur political activism and affect veterans’ political reintegration into civilian life, (2) the contribution of the data to the debate surrounding veteran activism, (3) emerging directions in the treatment of moral injury through political participation and social activism and, (4) an overview of the study’s limits and impact on future research.

### Moral injury, political activism, and civilian reintegration: the role of mediating narratives

Interviewees described several types of PMIEs that confirm the distinctions among self-perpetrated events, other-perpetrated events, and betrayal. Table 2 summarizes these distinctions and associated attributes. When events are self-perpetrated, the morally culpable agent is the active combatant. In other perpetrated acts, the culpable others were often commanders or comrades whose actions aroused feelings of betrayal or shame among some interviewees. Sometimes, interviewees faced social ingratitude or hostility for their actions. In these cases, feelings of betrayal arose from interviewees’ feelings that society failed to meet their expectations. Some interviewees also revealed how the culpable “others” were enemy agents who abused civilian immunities by recruiting human shields or using medical vehicles for military purposes. Such tactics placed the combat soldiers in an impossible situation by forcing them into morally transgressive behavior or rendering it impossible to complete their mission successfully. In each case, returning soldiers felt compelled to defend themselves in the face of criticism that some interpreted as betrayal. The pivotal role of culpable enemy agents is a significant finding that broadens the scope of PMIEs. Second, the data highlight two distinct humanitarian and national security narratives veterans use to interpret potentially morally injurious events. The humanitarian perspective is typical of morally injurious events recorded to date. In contrast, enemy-perpetrated events dominate the national security narrative and offer a singular contribution to the typology of moral injury.

**Table 2:** Summary of findings: moral injury and political activism.

PMIE	Narrative	Emotions	Culpable party	Activism
Self-perpetrated	Humanitarian	Shame, guilt	In-group: self or compatriots	Personal amends Conscientiousness raising
	National security	Anger, frustration	Out group: enemy, international community	Neutral and offensive information advocacy Patriotic education
Other-perpetrated/betrayal	Humanitarian	Anger	In-group: self or compatriots	Public protest
	National security	Anger, frustration	Out group: enemy, international community	Neutral and offensive information advocacy Patriotic education

Despite the novelty and significance of a dual-narrative interpretation of PMIEs, one may ask whether the national security narrative documents moral distress at all. This question deserves a brief comment. Because veterans adopting the national-security paradigm often place moral responsibility for transgressive acts on an enemy outgroup, one may cogently ask whether they perceive any moral transgression if veterans assign culpability to the other side. In their interviews, these activists suggest that the ethical dilemmas they experienced during their military service were not associated with their own actions. Instead, they were imposed by their enemies.

Blaming the other side suggests a process of moral disengagement whereby agents dissociate themselves, undermine the severity of their actions, or dehumanize the victim to resolve a moral dilemma they experience (62). Yet, it became evident during the interviews that the ethical dilemmas they experienced were not entirely resolved despite moral disengagement. Interviewees claimed to have acted congruently with their moral beliefs. However, these experiences still motivated their political activity, indicating that assigning culpability to the enemy does not necessarily resolve the moral conflicts soldiers experience when subject to outgroup-initiated PMIEs. Instead, such experiences may trigger anger and frustration that activists alleviate through political participation and social activism.

Finally, the data document very different ranges of emotions and subsequent political activism associated with each narrative and PMIE. Members of each group sought activities as part of a personal growth process following traumatic experiences that triggered shame, guilt, anger, and frustration. Humanitarian activists sought to alleviate these feelings, particularly shame and guilt, by demonstrating a commitment to human rights and social justice. On the other hand, national security-minded activists responded to anger and frustration with an urgent need to defend their actions and reinforce their patriotism. Factors explaining one’s preference for one narrative or another to explain morally challenging events may turn on such antecedent factors as political ideology, religiosity, culture, social consciousness, and personality traits. The interviews outline an underlying psychological mechanism that translates moral injury into political activism whereby differing narratives characterized by in-group or out-group orientations mediate potentially morally injurious events and help explain ensuing types of social and political activism as veterans return to civilian life.

The data in this study suggest alternative paradigms of moral injury that may not be sensitive to the standard diagnostic instruments (such as the MISS), one that may compel clinical researchers to develop additional moral injury evaluation instruments. Investigating the antecedent conditions of narrative building remains the topic of future study.

### Veteran activism

The benefits of offering veterans amends-making plans to expiate guilt and shame conform with this study’s results. The results also reinforce the published data corroborating the dominant emotions behind veteran activism (guilt, anger, and frustration) and the ensuing forms of political participation (protest) and social activism (volunteerism and education). While earlier studies differentiated between amends making (e.g., among soldiers returning from Afghanistan) and aggressive political protest (e.g., among Black veterans returning from Vietnam) (39–41, 50, 49), this study fleshes out two distinct narratives that help explain prior findings and serve as a starting point to study veteran activism further.

This study also offers a starting point to reflect on the role of veteran activism in a democratic society. When soldiers go to war, their compatriots expect a reckoning of sorts for killing in war, and postwar civil society is replete with ceremonies, medals, and customs to recognize combatants’ contribution and sacrifice, and assuage their guilt (63). It was Shay’s view that soldiers overcome with moral anguish from betrayal would develop such extreme distrust in state institutions that they would eschew democratic participation. Our study challenges this view. Soldiers betrayed by the state come back full of anger and indignation and dive into political protest. But this is not all. Shay did not consider that soldiers morally injured by self-perpetrated violations of humanitarian norms would channel their guilt and shame into social activism, volunteerism, and consciousness-raising to make amends for their actions. Nor did he or the psychologists treating moral injury leave room for soldiers whose moral anguish stems from the actions of their enemies whom they feel compel them to compromise their ethical values in defense of their country. These soldiers direct their anger and frustration into advocacy and education to defend their actions.

As the moral injury and moral distress resulting from combat gain growing recognition, compelling questions arise about how discharged service personnel reintegrate into civilian life. While researchers and clinicians take due note of how morally injurious combat experiences affect soldiers’ personal and family life, little research examines how combat trauma impacts a community’s political life and discourse. More than 3 million American soldiers supported the fighting in Iraq and Afghanistan during 20 years of warfare (64). In October 2023, Israel mobilized more than 300,000 reservists to augment its standing army (65). Many millions fight in other nations and other wars. As they return to the political arena, some veterans undertake intense social activism and political participation, and their impact reverberates beyond their numbers. They bring diverse political ideologies that direct their efforts toward numerous forms of social repair. Some seek to remedy the material harms of war directly, while others look to repair the reputational harms war has brought to their community. Together, these camps, both distinct and overlapping, one seeking amends, the other justification, cannot but enrich postwar discourse that may strongly influence a nation’s decisions to undertake future wars.

### Treatment for moral injury and combat-related moral distress

The central role of guilt and shame as motivators of social activism emerges when one considers the therapeutic value of social activism to help veterans by enabling post-traumatic growth (PTG). Facilitated by social activism and other interventions, PTG highlights the perceptual changes an individual undergoes within himself, his philosophy of life, and his relationships with others following a traumatic experience (66). PTG includes various phases wherein a person regains a new meaning to their life, self-reliance, and cognitive transformations that shift their consciousness from viewing themselves as a victim to viewing themselves as a praiseworthy survivor. Social activism, charitable work, and community volunteerism are intense among some veterans (67), and evidence suggests that these activities improve PTG and strengthen resilience among veterans and other trauma-affected populations (68–70). These findings are echoed in Schrader’s (51) interview-based research that shows how different kinds of activism, including anti-government, environmental, and social justice activism, alleviated the trauma soldiers experienced during their deployment in Iraq and Afghanistan. However, at other times, trauma may encourage people to seek reconciliation or redemption through social contribution to what’s “good,” permitting veterans to regain a sense of humanity by contributing to society and defying the inhumanity of war. Or it may also be the case that other, more conservative, veterans act on the pride and purpose of their service to challenge domestic and international criticism and denigration. In either case, political participation and social activism offer potentially effective means to mitigate the effects of moral injury and combat trauma. They remain a topic of further investigation.

### Limitations and future directions

Although limited, the interview data encourage additional hypothesis testing through large survey experiments [e.g., (71)]. The data in this study help explain how combat trauma and potentially morally injurious events inform and motivate politically and socially active veterans. A broader survey experiment can tell us the extent to which morally injured veterans engage in activism and enrich the democratic process. The data also support the identification of amends-making with the protest activism and volunteerism of veterans adopting a humanitarian narrative of PMIEs. At the same time, the data also highlight the tendency of betrayed veterans to adapt their activism to the source of their betrayal, whether of the humanitarian or national security bent. In this way, the data suggest a mechanism that links interpretations of PMIEs to political participation and enriches our understanding of veteran activism. Following armed conflict, nations may ignore the lessons of war or bring them to the center of public discourse. When vibrant democracies choose to engage, the voices of veteran activists are indispensable.

## Data availability statement

The raw data supporting the conclusions of this article will be made available by the authors, without undue reservation.

## Ethics statement

The studies involving humans were approved by Ethics Committee of the University of Haifa. Approval number 447/19. The studies were conducted in accordance with the local legislation and institutional requirements. The participants provided their written informed consent to participate in this study. Written informed consent was obtained from the individual(s) for the publication of any potentially identifiable images or data included in this article.

## Author contributions

AL: Investigation, Writing – original draft, Writing – review & editing, Conceptualization. MG: Investigation, Writing – original draft, Writing – review & editing.

## Footnotes

^1^These scores were lower than the average score of 50 in Koenig et al.’s (53) larger sample (*n* = 427). However, it is difficult to compare a small sample of politically active veterans with a larger sample of veterans and active-duty personnel with symptoms of PTSD, anxiety, and depression, among whom the lack of community involvement was moderately associated with moral injury. Items 6, 7 and 10 were reversely coded when calculating the final MISS score.
